# In Vitro Investigation of the Anticancer Properties of Ammodaucus Leucotrichus *Coss. & Dur.*

**DOI:** 10.3390/ph15121491

**Published:** 2022-11-30

**Authors:** Monia Lenzi, Eleonora Turrini, Elena Catanzaro, Veronica Cocchi, Alessandra Guerrini, Patrizia Hrelia, Sofia Gasperini, Claudio Stefanelli, Mohamed Lamin Abdi Bellau, Valentina Pellicioni, Massimo Tacchini, Giulia Greco, Carmela Fimognari

**Affiliations:** 1 Department of Pharmacy and Biotechnology, *Alma Mater Studiorum*-Università di Bologna, via San Donato 15, 40127 Bologna, Italy; 2 Department for Life Quality Studies, *Alma Mater Studiorum*-Università di Bologna, Corso d’Augusto 237, 47921 Rimini, Italy; 3 Cell Death Investigation and Therapy (CDIT) Laboratory, Department of Human Structure and Repair, Ghent University, 9000 Ghent, Belgium; 4 Pharmaceutical Biology Lab., Research Unit 7 of Terra&Acqua Tech Technopole Lab., Department of Life Sciences and Biotechnology, University of Ferrara, Piazzale Luciano Chiappini 3, 44123 Ferrara, Italy; 5 Sahrawi Refugee Camps, Tindouf 37000, Algeria; 6 Department of Chemistry “Giacomo Ciamician”, *Alma Mater Studiorum*-Università di Bologna, via Selmi 2, 40126 Bologna, Italy

**Keywords:** *Ammodaucus leucotrichus*, perillaldehyde, antimutagenesis, apoptosis, cancer cells, cytotoxicity, DNA damage

## Abstract

Little is known about the pharmacological activity of *Ammodaucus leucotrichus Coss. & Dur*., a small annual species that grows in the Saharan and sub-Saharan countries. In the present study, we investigated whether the standardized ethanolic extract of *A. leucotrichus* fruits and R-perillaldehyde, a monoterpenoid isolated from *A. leucotrichus* fruits, are able to affect different processes involved in different phases of cancer development. In particular, we explored their genoprotective, proapoptotic, antiproliferative, and cytodifferentiating potential on different human cell models. We analyzed the genoprotective and proapoptotic activity on human lymphoblast cells (TK6) using the micronucleus test, and the cytodifferentiation effects on human promyelocytic cells (HL60) through the evaluation of different markers of differentiation forward granulocytes or monocytes. The results showed that the extract and perillaldehyde were able to induce apoptosis and protect from clastogen-induced DNA damage. To our best knowledge, this is the first report on the ability of *A. leucotrichus* and perillaldehyde to induce apoptosis and protect DNA from the toxicity of different compounds. Data reported in this work are the starting point for their pharmacological use. Going forward, efforts to determine their effects on other events associated with cancer development, such as angiogenesis and metastasization, will provide important information and improve our understanding of their potential in cancer therapy.

## 1. Introduction

Current pharmacological approaches for cancer treatment include the use of a plethora of chemotherapeutic drugs, which are characterized by multiple adverse and toxic effects [1]. Recently, the use of botanicals in the oncological area was extensively explored due to their complex content of phytochemicals interacting with multiple molecular targets and exhibiting a multitarget effect. The multitarget nature of botanicals can represent a very useful approach to facing the biological complexity of tumors [2,3,4,5,6,7,8,9]. Although 60% of antiblastic agents are derived from botanicals and other natural resources, there are still many plants endowed with anticancer potential, but they have not yet been fully investigated.

*Ammodaucus leucotrichus Coss. & Dur.* (Apiaceae), known in Algeria as “Kammûn es-sofi”, is an aromatic small annual species that grows in the Saharan and sub-Saharan countries of North Africa, including Morocco, Algeria, and Tunisia extending to Egypt and tropical Africa [10]. This plant is glabrous, 10–12 cm high, with erect and finely striated stems, branched from the base. The leaves are fleshy and finely divided. The small flowers with five free white petals are grouped in umbels of two to four branches. The fruit is a diachene, 8–10 mm long, covered with dense and white hairs. The species usually flowers between February and April. The plant grows spontaneously in wadis, on sandy-gravelly soils in arid conditions where the annual rainfall does not exceed 100 mm. To the best of our knowledge, there are no data on its propagation and conservation; it is currently not cultivated [11,12]. The fruits of *A. leucotrichus*, harvested when ripe, are used in traditional medicine as a remedy for several diseases including rheumatism, asthma and gastro-intestinal pain [13,14], nausea, vomiting, dehydration, excessive production of urine [11,15], and cardiac diseases [16]. Moreover, the plant’s fruits are used as cooking ingredients, particularly as substitutes for cumin [17,18]. 

Although *A. leucotrichus* plays a major role in the traditional medicine of North African countries for the treatment of many diseases, few experimental studies have explored its pharmacological effects. Scientific evidence demonstrates that the fruit aqueous extract of *A. leucotrichus* exerts a protective effect against urinary diseases, such as lithiasis, where it blocks the growth of calcium oxalate monohydrate crystals [19]. Moreover, two recent studies investigated its antidiabetic potential. Oral treatment with the fruit water extract of *A. leucotrichus* decreased plasma glucose levels, improved glucose tolerance, and protected the liver tissues of diabetic rats [20]. Inhibition of α-amylase and α-glucosidase activity and reduced intestinal glucose absorption have also been reported for a fruit aqueous extract in vitro and in diabetic rats [21]. Its essential oil exhibited a potent antibacterial effect against *Staphylococcus aureus*, *Escherichia coli*, and *Klebsiella pneumonia* [22,23], and was endowed with antioxidant activity [23]. Antioxidant as well as anti-glycation activity has been recently reported also for seeds hydroalcoholic [24] and fruit aqueous extracts [25] obtained from *A. leucotrichus*.

The essential oil of *A. leucotrichus* has been found to contain R-perillaldehyde and limonene [10,26,27,28,29]. In our previous research, the decoction and ethanolic extract were found to contain R-perillaldehyde and ammolactone-A [29]. Perillaldehyde and limonene are commercially used as flavors and preservatives in foods and perfumes [30,31]. Moreover, some studies reported antidepressant effects in stress-induced depression-like model mice and anticancer effects for perillaldehyde [32,33,34] and Ras-independent anticancer effects on PANC-1 pancreas carcinoma cells for limonene [35].

The current study aimed to identify whether *A. leucotrichus* exhibits significant anticancer activity and modulates different processes involved in different phases of cancer development. In particular, we explored its genoprotective, proapoptotic, antiproliferative, and cytodifferentiating potential on different human cell models. We used TK6 cells, as recommended by the OECD guidelines [36], to analyze their genoprotective abilities by the micronuclei (MNi) test. On the same model, we investigated its apoptotic and antiproliferative effects. HL60 cells were used to explore their cytodifferentiative potential based on their ability to differentiate forward the macrophagic and/or granulocytic lineage [37].

## 2. Results and Discussion

### 2.1. Qualitative and Quantitative Characterization of Ammolactone-A and R-Perillaldehyde in GC-MS and HPLC-DAD

**Figure 1:**
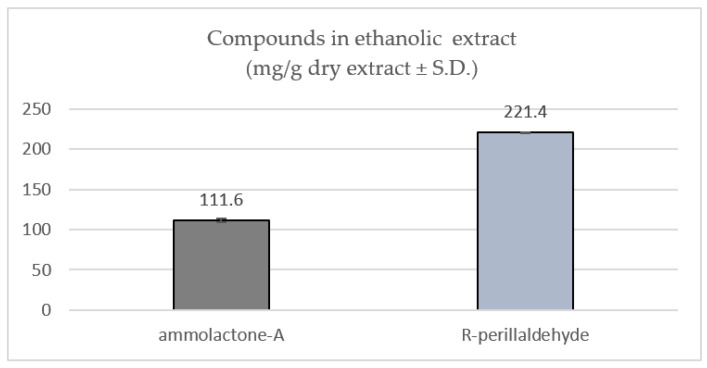
HPLC-DAD quantitative analysis of *A. leucotrichus*.

As reported in our earlier published research [29], the ethanolic extract of *A. leucotrichus* fruits showed an extraction yield of 6.58 ± 0.07% (*w*/*w*) and two predominant peaks in HPLC-DAD: ammolactone-A and R-perillaldehyde. These two molecules were previously isolated, characterized by GC-MS and NMR experiments, and quantified in the ethanolic extract [29], as shown in Figure 1.

### 2.2. A. Leucotrichus Extract and Perillaldehyde Exhibit Cytotoxicity on Different Cancer Cell Lines

The cytotoxic effects of *A. leucotrichus* extract and perillaldehyde were analyzed on four different human cell lines, two from hematological tumors and two from solid tumors: lymphoblast cells (TK6), promyelocytic cells (HL60), colorectal adenocarcinoma cells (DLD1), and neuroblastoma cells (SHSY5Y). The extract decreased cell viability in a dose-dependent manner in all analyzed cell lines after 24–26 h treatment. The inhibitory concentrations causing 50% of cell toxicity (IC_50_) values were calculated and reported below (Table 1). 

No significant difference was observed among the IC_50_ values on the tested cell lines for the extract. Similar results were recorded for perillaldehyde (data not shown). Thus, the following studies were performed on hematological cancer cells, which are easier to cultivate and analyze by flow cytometry than solid cell lines.

### 2.3. A. Leucotrichus Extract and Perillaldehyde Do Not Increase the Frequency of MNi and Induces Apoptosis on TK6 Cells

#### 2.3.1. Short-Term Treatment

**Figure 2:**
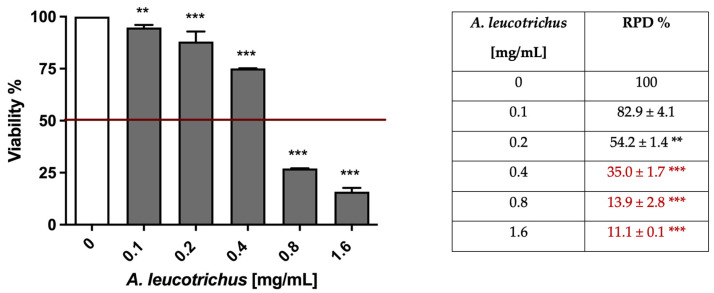
Cell viability (**left**) and RPD (**right**) on TK6 cells after 3 h treatment with *A. leucotrichus* extract followed by 23 h of recovery in the complete medium at the indicated concentrations compared to the untreated cultures (0). In red are indicated the RPD values not in compliance with the OECD recommended threshold. ** *p* < 0.01; *** *p* < 0.001 vs. 0.

As established by OECD for the mutagenic analysis [36], MNi frequency was measured after a short-term treatment of 3 h followed by 23 h of recovery and after a long-term treatment of 26 h [20]. We preliminary checked whether cellular viability and proliferation percentages respected the threshold (equal to 55 ± 5%) established by OECD for the mutagenic analysis [36]. Starting from 0.1 mg/mL, *A. leucotrichus* dose-dependently reduced cell viability (Figure 2, left panel) and proliferation, as assessed by the RPD (relative population doubling) % (Figure 2, right panel). In Figure 2 it can be seen how the cytotoxicity induced by *A. leucotrichus* complied with the OECD guideline threshold (represented by the red line) up to 0.4 mg/mL concentration. At the same time, using the RPD value, we observed that A. *leucotrichus* complied with the OECD threshold of up to a 0.2 mg/mL concentration (Figure 2, right panel).

**Figure 3:**
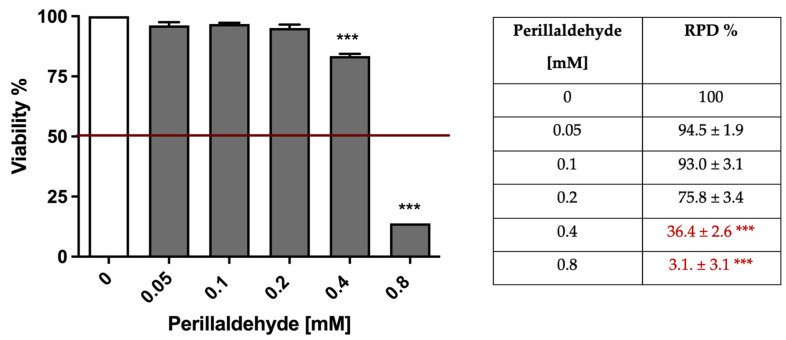
Cell viability (**left**) and RPD (**right**) on TK6 cells after 3 h treatment with perillaldehyde followed by 23 h of recovery in the complete medium at the indicated concentrations compared to the untreated cultures (0). In red are indicated the RPD values not in compliance with the OECD recommended threshold. *** *p* < 0.001 vs. 0.

The cytotoxicity induced by perillaldehyde complied with the OECD guideline threshold of up to 0.4 mM. At the same time, using the RPD value, perillaldehyde complied with the OECD threshold of up to 0.2 mM (Figure 3, right panel).

**Figure 4:**
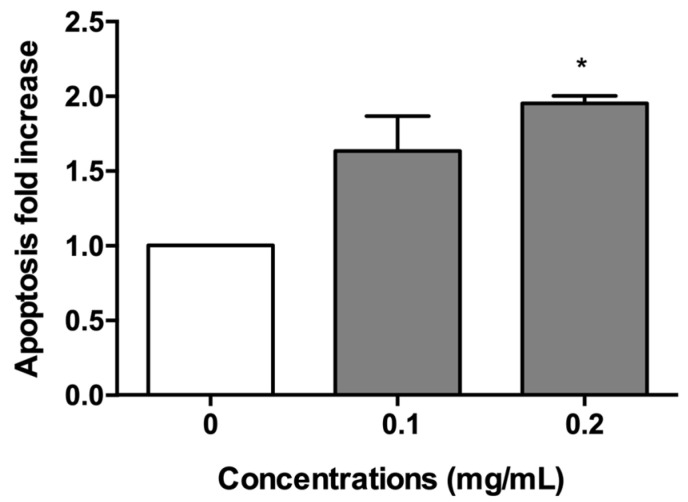
Apoptosis fold increase on TK6 cells after 3 h treatments with *A. leucotrichus* (0.1 and 0.2 mg/mL) followed by 23 h of recovery in the complete medium at the indicated concentrations compared to untreated cultures (0). * *p* < 0.05 vs. 0.

Subsequently, the induction of apoptosis was evaluated as a cell death mechanism to avoid the possible confounding effects of apoptotic bodies with MNi. In particular, the apoptosis fold increases reached almost a doubling in cultures treated with 0.2 mg/mL. Smaller increases were observed at 0.1 mg/mL (Figure 4).

For perillaldehyde, the apoptosis fold increases reached almost a doubling in cultures treated with 0.2 mM. Smaller increases were observed at 0.1 mM, while apoptosis resulted comparable to that measured in the concurrent negative control in cultures treated with perillaldehyde at 0.05 mM (data not shown).

To avoid the possible confounding effect of apoptotic bodies with MNi, the lowest concentrations tested (0.1 mg/mL for *A. leucotrichus* and 0.05 mM for perillaldehyde) were selected for the MNi frequency analysis.

**Figure 5:**
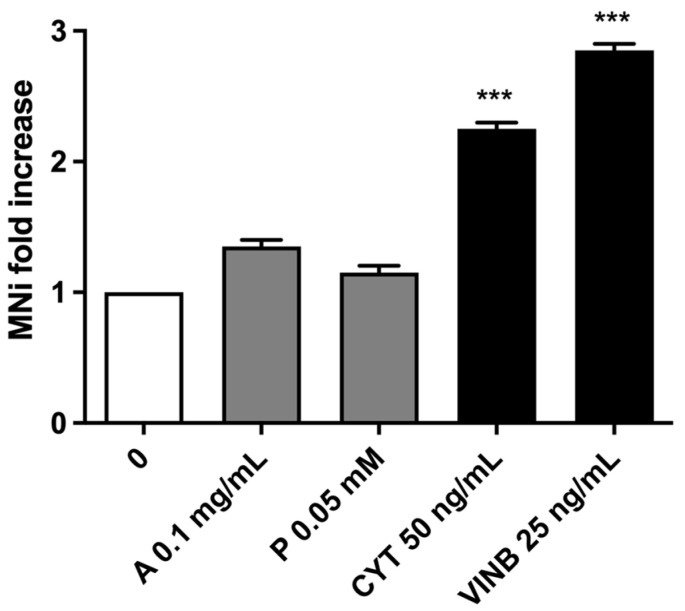
MNi fold increase on TK6 cells after 3 h treatments with *A. leucotrichus* (A), perillaldehyde (*p*), or positive controls (CYT, VINB) followed by 23 h of recovery in the complete medium at the indicated concentrations compared to the untreated cultures (0). *** *p* < 0.001 vs. 0.

As shown in Figure 5, *A. leucotrichus* and perillaldehyde did not induce a statistically significant increase in MNi frequency, while an increase equal to two or three times was detected in the cultures treated with cytosine arabinoside (CYT) or vinblastine (VINB), used as positive controls.

#### 2.3.2. Long-Term Treatment

Cell viability and proliferation were explored also after long-term treatment (26 h). Similarly to what we did for the short-time treatment, for the analysis of MNi we selected the concentration of 0.025 mg/mL for *A. leucotrichus* and 0.025 mM for perillaldehyde, which complied with the OECD guideline threshold on the basis of cytotoxicity, cytostasis, and apoptosis. Table 2 shows how the selected concentrations comply with the OECD threshold for cytotoxicity (viability above 55 ± 5%) and cytostasis (RPD above 55 ± 5%). Moreover, apoptosis levels of these treated cultures were comparable to that measured in the untreated cultures. Of note, from the analysis of cell-cycle progression after 26 h from treatment, we did not record a cell-cycle block at any tested concentration, but only an accumulation of cells in the sub-G1 phase (data not shown). The rise of cells in the sub-G1 phase indicates an increase in the percentage of cells with fragmented DNA, characteristic of dead cells [38].

Finally, no increases in MNi frequency were observed after *A. leucotrichus* or perillaldehyde treatment (Table 2). 

Mutagenicity is a mechanism strictly associated with carcinogenesis [39]. Since mutagenic events are irreversible and permanent, mutagenic compounds lack a threshold. This means that just one mutation in oncogenes can potentially give rise to a cancerous cell and that cancer risk is zero only if there is no exposure to a mutagenic agent [40]. 

As reported above in the phytochemical analysis, the extract of *A. leucotrichus* contains perillaldehyde. Perillaldehyde has an α,β-unsaturated aldehyde structure, which is electrophilic and can covalently bind to electron-rich macromolecules such as DNA through a 1,4-nucleophilic addition to generate DNA adducts [41]. The mutagenicity of perillaldehyde was systematically investigated using in vitro (Ames tests, MNi assay, HPRT mutation assay) and in vivo tests (MNi and comet assays, transgenic rodent gene mutation) [42]. Even if the genotoxic properties of perillaldehyde remain under dispute, in our experimental setting the extract and perillaldehyde are clearly non-genotoxic. 

### 2.4. A. Leucotrichus Extract and Perillaldehyde Modulate the Mutagenicity of Different Compounds

#### 2.4.1. Short-Term Treatment

**Figure 6:**
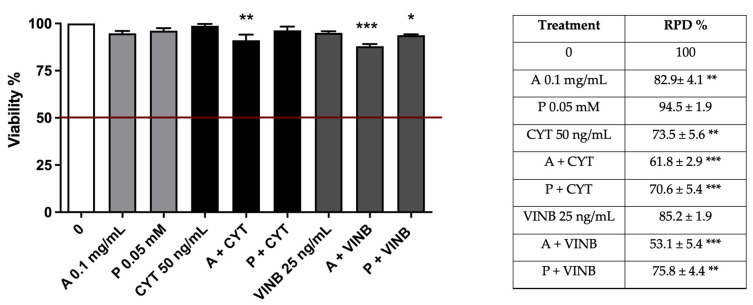
Cell viability (**left**) and RPD (**right**) on TK6 cells after 3 h treatments with *A. leucotrichus* (A) or perillaldehyde (P) followed by 23 h of recovery in the complete medium at the indicated concentrations in the presence or absence of CYT (clastogen) or VINB (aneugen) compared to the untreated cultures (0). * *p*< 0.05 vs. 0; ** *p*< 0.01; *** *p* < 0.001 vs. 0.

Once the toxicity and the mutagenicity of the extract and perillaldehyde were excluded at both treatment times, the study continued evaluating their possible antimutagenic activity against the known mutagens previously used as positive controls (CYT, VINB) after short- and long-term treatments. We preliminary evaluated the toxicity of the extract in association with CYT or VINB. A co-treatment of 3 h followed by 23 h of recovery in the complete medium was performed, and cytotoxicity, cytostasis, and apoptosis were checked before proceeding with the mutagenicity analysis. As shown in Figure 6, cell viability (left panel) and RPD values (right panel) were abundantly above the threshold established by the OECD guideline no. 487 at all the experimental conditions.

**Figure 7:**
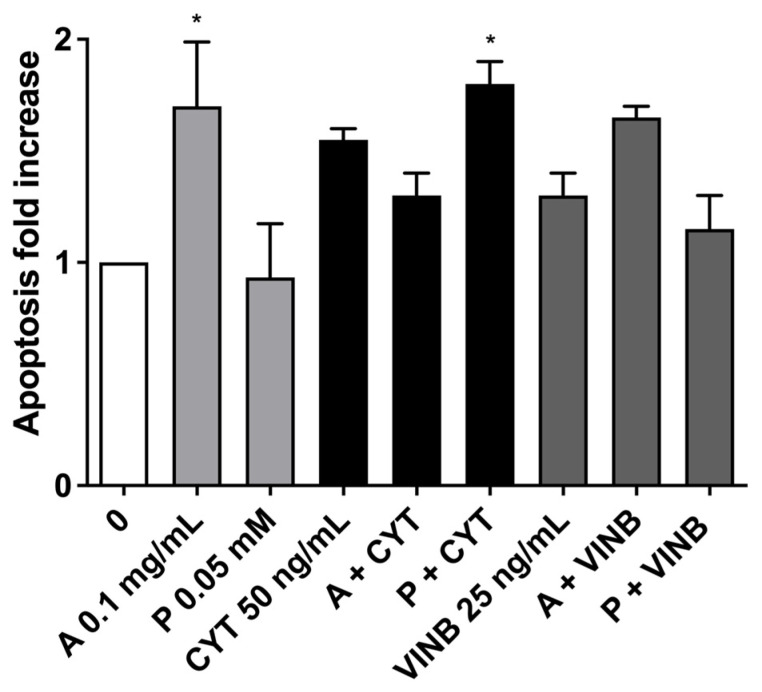
Apoptosis fold increase on TK6 cells after 3 h treatments with *A. leucotrichus* (A) or perillaldehyde (P) followed by 23 h of recovery in the complete medium at the indicated concentrations in the presence or absence of CYT (clastogen) or VINB (aneugen) compared to the untreated cultures (0). * *p*< 0.05 vs. 0.

On average, the apoptosis fold increase was smaller than a doubling for all the experimental conditions (Figure 7).

**Figure 8:**
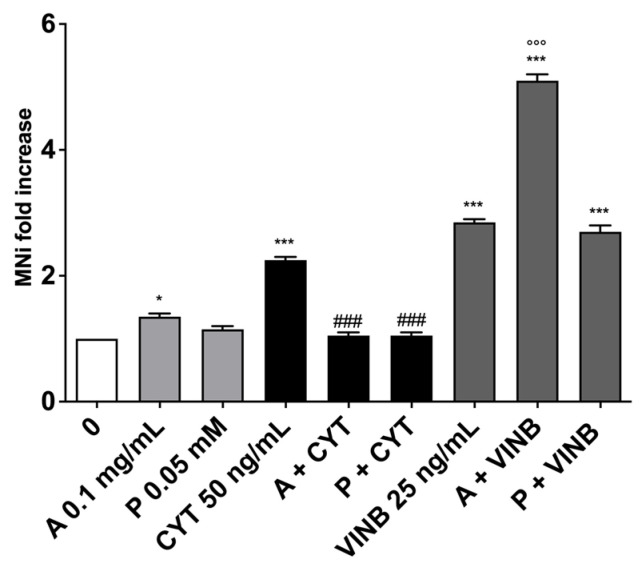
MNi fold increase on TK6 cells after 3 h treatments with *A. leucotrichus* (A) or perillaldehyde (P) followed by 23 h of recovery in the complete medium at the indicated concentrations in the presence or absence of CYT (clastogen) or VINB (aneugen) compared to the untreated cultures (0) and the relative positive controls. * *p* < 0.05; *** *p* < 0.001 vs. 0; °°° *p* < 0.001 vs. VINB; ### *p* < 0.001 vs. CYT.

Overall, the results obtained allowed us to proceed with the MNi frequency analysis, which demonstrated the *A. leucotrichus* extract’s ability to decrease the MNi frequency induced by CYT. On the contrary, the extract increased the MNi frequency when cells were co-treated with VINB (Figure 8). Analogously, perillaldehyde was more effective in preventing the DNA damage caused by CYT, while almost no differences in the MNi frequency were detected when in co-treatment with VINB (Figure 8).

#### 2.4.2. Long-Term Treatment

**Figure 9:**
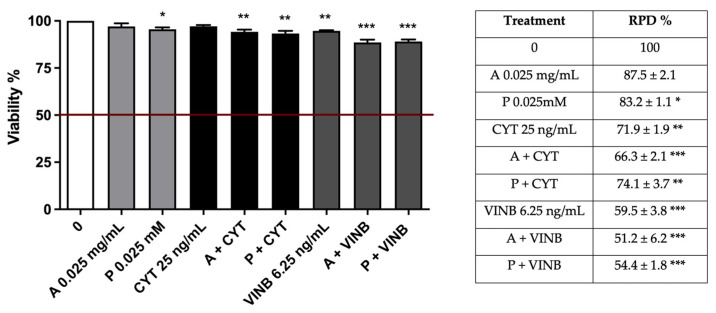
Cell viability (left) and RPD (right) on TK6 cells after 26 h treatments with *A. leucotrichus* (A) or perillaldehyde (P) at the indicated concentrations in the presence or absence of CYT (clastogen) or VINB (aneugen) compared to the untreated cultures (0). * *p*< 0.05 vs. 0; ** *p*< 0.01 vs. 0; *** *p* < 0.001 vs. 0.

The study concluded by evaluating the antimutagenic activity of *A. leucotrichus* and perillaldehyde at 26 h. Cytotoxicity, cytostasis, and apoptosis were preliminary checked to verify if they respected the established OECD thresholds at all the conditions tested (Figure 9). 

**Figure 10:**
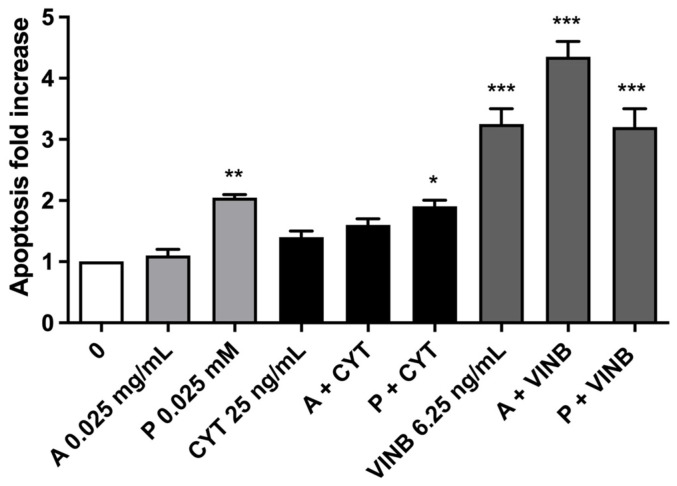
Apoptosis fold increase on TK6 cells after 26 h treatments with *A. leucotrichus* (A) or perillaldehyde (P) at the indicated concentrations in the presence or absence of CYT (clastogen) or VINB (aneugen) compared to the untreated cultures (0). * *p*< 0.05 vs. 0; ** *p*< 0.01 vs. 0; *** *p* < 0.001 vs. 0.

Figure 10 shows a three-fold increase in apoptosis in cells treated with VINB or VINB plus perillaldehyde, respectively, and a four-fold increase in cells treated with VINB plus *A. leucotrichus*, respectively. 

Therefore, once checked cytotoxicity and cytostasis, the study ended by evaluating the antimutagenic activity of *A. leucotrichus* and perillaldehyde after 26 h treatment.

**Figure 11:**
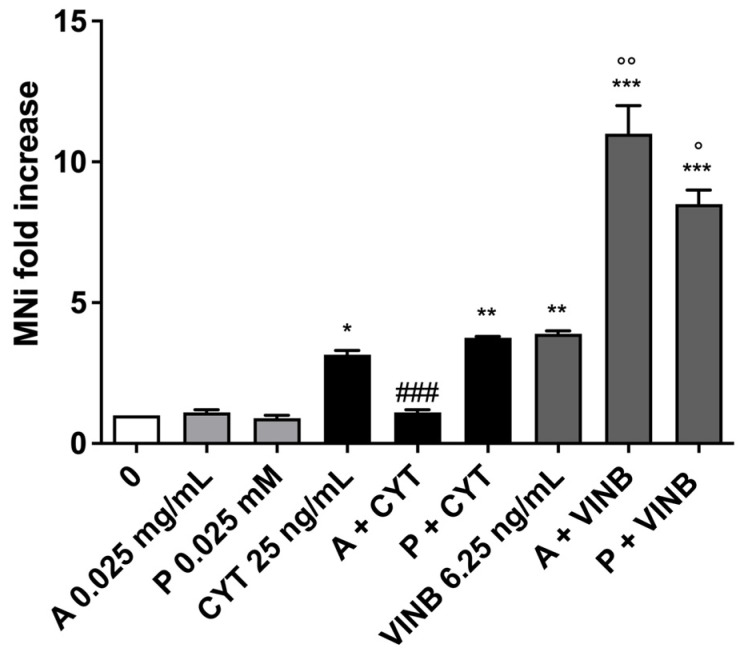
MNi fold increase on TK6 cells after 26 h treatments with *A. leucotrichus* (A) at the indicated concentrations in the presence or absence of CYT (clastogen) or VINB (aneugen) compared to the negative control (0) and the relative positive controls. * *p*< 0.05; ** *p* < 0.01; *** *p* < 0.001 vs. 0; ° *p*< 0.05 vs. VINB; °° *p* < 0.01 vs. VINB; ### *p* < 0.001 vs. CYT.

We recorded a decrease in the MNi frequency only in the cultures co-treated with *A. leucotrichus* plus CYT, while an increase occurred in cells treated with A. *leucotrichus* plus VINB (Figure 11). In the co-treatments with perillaldehyde, a slight increase in MNi frequency was observed with CYT and a greater increase with VINB (Figure 11).

Clastogens are DNA-reactive agents able to cause chromosome breaks or rearrangements; aneugens affect cell division and the mitotic spindle apparatus and give rise to the loss or gain of whole chromosomes. Unlike clastogens, aneugens exhibit well-established non-linear dose–responses, thus they have a threshold [43]. The different mode of action of clastogens and aneugens is important for hazard and risk assessment [44]. In our experimental settings, we explored the protective ability of *A. leucotrichus* extract and perillaldehyde against the clastogenic compound CYT and the aneugenic compound VINB. On the whole, our results indicate that the addition of *A. leucotrichus* decreased the number of MNi induced by CYT, whereas both the extract and perillaldehyde increased the number of MNi induced by VINB. Bearing in mind that the addition of the extract or perillaldehyde significantly increased the proapoptotic activity of VINB, the increase in the VINB-induced MNi number calls into question whether the response seen is a function of an additive mutagenic effect or simply a toxic response. To better understand the reason for the different effect of the extract and perillaldehyde against clastogens and aneugens, additional experiments should be designed including a greater number of mutagens, thus defining whether the effects reported in our study is peculiar for VINB or is common to other aneugenic compounds. 

### 2.5. A. Leucotrichus Extract and Perillaldehyde Do Not Affect the Differentiation of HL60 Cells

The engagement of differentiation represents an alternative method compared to apoptosis for terminating the malignant proliferation of cancer cells. Indeed, differentiated cells have lower levels of survival-maintaining factors than their progenitors, the great immaturity of which contributes to their apoptosis resistance [45]. We first analyzed the ability of the extract to induce terminal differentiation toward granulocytes and/or macrophages of HL60 cells, which are predominantly promyelocytes. 

The analysis of cytodifferentiation has to be performed at concentrations where cell viability is higher than 80% after 72 h treatment with the tested compound [46]. *A. leucotrichus* was tested at concentrations up to 0.1 mg/mL, where cell viability was higher than 80% (data not shown). 

**Figure 12:**
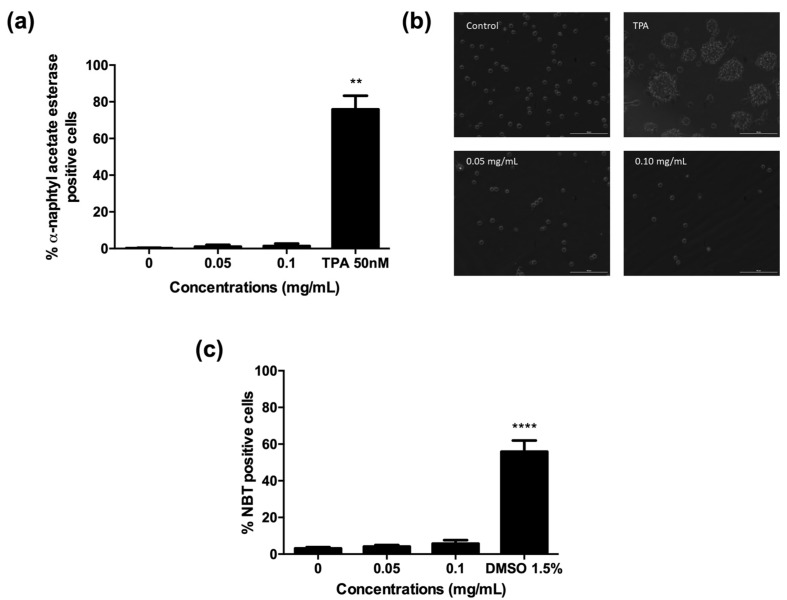
Effects of *A. leucotrichus* (0.05 and 0.1 mg/mL) on HL60 cell differentiation as analyzed by α-naphthyl acetate esterase activity (**a**); cell adhesion, magnitude 20x (**b**); NBT reducing activity (**c**). 12-O–tetradecanoylphorbol 13-acetate (TPA) and dimethyl sulfoxide (DMSO) were used as positive controls. ** *p* < 0.01; **** *p* < 0.0001 vs. untreated cultures (0).

All the tests performed to assess the cytodifferentiation ability of *A. leucotrichus* revealed the absence of differentiation forward granulocytes or monocytes. In particular, no significant increase was observed in α-naphthyl acetate esterase positive cells, except for the positive control 12-O–tetradecanoylphorbol 13-acetate (TPA) 50 nM (Figure 12a). Accordingly, no increase in the number of adherent cells was observed after treatment with the extract, excluding differentiation into monocytes/macrophages (Figure 12b). Indeed, the images show that even fewer cells than the untreated cultures are anchored to the plastic support after treatment with the extract and no morphological changes were observed compared to TPA used as the positive control, where monocyte/macrophage morphology is evident (Figure 12b). Moreover, the nitro blue tetrazolium (NBT) reduction assay did not reveal any differentiation forward granulocyte/monocyte lineage of HL60 cells, but a significant differentiation was recorded after treatment with dimethyl sulfoxide (DMSO) 1.5% (Figure 12c) used as the positive control, which supports a proper test performance. 

A long-held dogma in acute myeloid leukemia is that primitive myeloblast cells are characterized by a blockage of cell differentiation. Accordingly, the treatment of acute myeloid leukemia and, in particular, acute promyelocytic leukemia involves the use of drugs able to induce terminal differentiation, such as all-trans retinoic acid and arsenic trioxide [47]. As an example, treatment of HL60 cells with retinoic acid can induce terminal differentiation to neutrophil-like cells, which is followed by an increase in the number of cells with morphological characteristics of apoptosis [48]. No other leukemia differentiation drugs are clinically available. The discovery of new differentiation compounds is therefore important for leukemia patients. In our experimental conditions, *A. leucotrichus* extract was not able to induce terminal differentiation of HL60 cells. Similar results were recorded for perillaldehyde (data not shown). Thus, their ability to induce apoptosis is clearly independent of cytodifferentiation.

## 3. Materials and Methods

### 3.1. Reagents

CYT, ethylenediaminetetraacetic acid (EDTA), fetal bovine serum (FBS), L-glutamine (L-GLU), Nonidet, penicillin-streptomycin solution (PS), phosphate buffer saline (PBS), potassium chloride, potassium dihydrogen phosphate, Roswell Park Memorial Institute (RPMI) 1640 medium, water BPC grade, sodium chloride, sodium hydrogen phosphate, VINB were purchased from Merck, Darmstadt, Germany. Guava Nexin Reagent and Guava ViaCount Reagent were purchased from Luminex Corporation, Austin, TX, USA. RNase A and SYTOX Green were purchased from Thermo Fisher Scientific, Waltham, MA, USA. 

### 3.2. Plant Material

The fruits of *A. leucotrichus* were collected from a wild population of plants at Bir Lehlu (coordinates: 26°20′58″ N 09°34′32″ W, Western Sahara), in March 2016. Plant material collection was carefully performed in order not to damage the wild population and obtaining a representative sample from no less than 10 plants. The sample authentication was performed by dr. Mohamed Lamin Abdi Bellau and prof. Alessandra Guerrini, according to the IUCN Centre for Mediterranean Cooperation [11]. The voucher specimen (code no. AMM.022.001) is stored in the Herbarium of the University of Ferrara (Italy). The plant material was dried at room temperature for 15 days.

### 3.3. Preparation of Ethanolic Extract and Chemical Characterization

*A. leucotrichus* ethanolic extract was prepared and chemically characterized according to the methods previously described [29].

### 3.4. Cell Cultures and Treatments

DLD1 (human colon adenocarcinoma cells, ATCC-CCL-221) and SHSY5Y (human neuroblastoma cells, ATCC-CRL-2266) were purchased from LGC Standard (LGC Group, Middlesex, UK). HL60 cells (human promyelocytic leukemia, BS TCL25) were obtained from Istituto Zooprofilattico of Brescia (Brescia, Italy). TK6 cells (human lymphoblastoid, #95111735) were purchased from Merck (Darmstadt, Germany). TK6 and DLD1 were propagated in RPMI 1640 supplemented with 10% heat-inactivated FBS, 1% l-glutamine solution 200 mM, and 1% penicillin (10,000 units)/streptomycin (10 mg/mL) solution. HL60 were cultured in the same culture medium with the only exception of 20% inactivated FBS. SHSY5Y cells were propagated in DMEM high glucose (Gibco, Thermo Fisher Scientific) supplemented with 10% FBS, 1% l-glutamine 200 mM, and 1% penicillin/streptomycin. All cells were incubated at 37 °C with 5% CO_2_ in the humified atmosphere. To obtain exponential growth and avoid spontaneous differentiation, the culture density of HL60 cells was never allowed to exceed 1.0 × 10^6^ cells/mL. To maintain exponential growth and considering that the time required to complete the cell cycle is about 13 h, TK6 cultures were divided every three days in fresh medium and the cell density did not exceed the critical value of 9 × 10^5^ cells/mL. 

Cells were treated with *A. leucotrichus* extract or R-perillaldehyde at 0–1.6 mg/mL and at 0–0.8 mM, respectively.

### 3.5. Flow Cytometry

All flow cytometric analyses were performed by the Guava EasyCyte 6-2L or EasyCyte 5HT flow cytometer (Luminex Corporation, Austin, TX, USA). At least 10,000 events were evaluated for each analyzed sample.

### 3.6. Cytotoxicity Analysis

After treatment with *A. leucotrichus*, the percentage of viable cells was evaluated by the Guava ViaCount Reagent (Merck), containing 7-AAD or propidium iodide (PI) for suspension cells or the MTT [3-(4,5-dimethylthiazol-2-yl)-2,5-diphenyltetrazolium bromide] assay for adherent cells. The viability percentage recorded in the treated cultures was normalized on that recorded in the concurrent negative control cultures, considered equal to 100%. IC_50_ was calculated by interpolation from the dose–response curve.

### 3.7. Cytostasis Analysis

The number of cells seeded at time 0 (initial cell number) and that measured at the end of the treatment time (post-treatment cell number) were evaluated using the Guava ViaCount Reagent. From those values, we derived the population doubling (*PD*):

$$PD=\left[\log\left(post-treatment\;cell\;number÷\mathrm{initial}\;\mathrm{cell}\;\mathrm{number}\right)\right]÷2$$

The *PD* obtained in the control cultures was compared to that measured in the treated cultures, obtaining the *RPD* value. *RPD* was used to measure cell proliferation and to check that a substantial proportion of the cells have undergone division at the end of treatment time. *RPD* was calculated with the following formulas:

(1) $$RPD=\frac{PD\;in\;treated\;cultures}{PD\;in\;control\;cutures}\;\times \;100$$

For the analysis of cell-cycle progression, after 24 h from treatment, cells were fixed and permeabilized with 70% ice-cold ethanol. After 30 min, cells were washed with PBS 1x and incubated with Guava Cell Cycle Reagent (Merck), containing PI for 30 min in the dark. The analysis of samples was performed via flow cytometry.

### 3.8. Apoptosis Analysis

After treatment, cells were incubated with 100 µL of Guava Nexin Reagent (Merck), containing 7-AAD and annexin V-phycoerythrin, for 20 min at room temperature in the dark, and analyzed by flow cytometry. The apoptotic cell percentage recorded in the treated cultures was normalized on that recorded in the concurrent negative cultures, considered equal to 1, and expressed as apoptotic fold increase [49].

### 3.9. MNi Frequency Analysis

MNi frequency was evaluated in cultures treated with *A. leucotrichus* or perillaldehyde with or without the clastogen CYT and the aneugen VINB, used as positive controls [36]. The analysis of the MNi frequency was performed using an automated protocol published by Lenzi et al. [50] already successfully employed in different previous studies [51,52,53,54].

Briefly, at the end of the treatment time, cells were collected, lysed, and stained with SYTOX Green. The discrimination between nuclei and MNi was performed based on the different sizes analyzed by forward scatter (FSC) and the different intensities of green fluorescence. The MNi frequency, calculated as the number of MNi per 10,000 nuclei deriving from living and proliferating cells and recorded in treated cultures at all concentrations tested, was normalized on those recorded in the untreated cultures.

### 3.10. Analysis of Cell Differentiation

The cytodifferentiation potential was evaluated in HL60 cells treated with *A. leucotrichus* or perillaldehyde for 72 h, as previously described [55]. After incubation, cells were analyzed for the presence of markers or morphological features of differentiation along the granulocytes and/or macrophages. The following assays were performed: α-naphthyl acetate esterase activity, indicative of differentiation forward monocytic/macrophage lineage; NBT—reducing activity, which indicates differentiation along both monocytic and granulocytic lineage; and adherence to the plastic substrate to evaluate macrophagic differentiation. 

#### 3.10.1. α-Naphthyl Acetate Esterase Activity

The activity of α-naphthyl acetate esterase was assessed using the cytochemical 91-A kit [56], according to the manufacturer’s instructions (Merck). Cells were examined using the inverted microscope Nikon ECLIPSE Ts2, distinguishing dark/positive cells from light/negative cells. At least 200 cells from two different operators were examined for each experiment.

#### 3.10.2. NBT Reduction Assay

The NBT assay evaluates the ability of a compound to generate superoxide when exposed to TPA [57]. Briefly, after 72 h treatment cells were collected and treated with a freshly prepared solution of 0.2% NBT (Merck) and 200 ng/mL TPA (Merck) in PBS 1x. After 30 min incubation at 37 °C, the reaction was stopped by placing the samples on ice for 5 min. Cells were then prepared for analysis via an inverted microscope Nikon ECLIPSE Ts2 on glass slides. Intracellular black–blue formazan deposits indicate NBT reduction, thus positive cells. At least 500 cells were examined for each experiment from two different operators. DMSO 1.5% (*v*/*v*) was used as the positive control. Results were expressed as percentages of positive cells over total cells.

#### 3.10.3. Adherence to the Plastic Substrate

After 72 h of treatment, the medium was removed and the non-adherent cells were gently removed, washing with PBS 1x. Dish-anchored cells were counted using the inverted microscope Nikon ECLIPSE Ts2 [37]. TPA 50 nM was used as the positive control.

### 3.11. Statistical Analysis

Results are expressed as the mean ± SEM of at least three independent experiments. Statistical analyses were performed using one-way or two-way ANOVA test and Dunnett or Bonferroni as post-test, using GraphPad InStat 6.0 version (GraphPad Prism, San Diego, CA, USA); *p* values below 0.05 were considered significant.

## 4. Conclusions

To our best knowledge, this is the first report on the ability of *A. leucotrichus* to induce apoptosis and protect DNA from the toxicity of different compounds. Data reported in this work are the starting point for its pharmacological use. Going forward, efforts to determine its effects on other events associated with cancer development, such as angiogenesis and metastasization, and to analyze its activity in association with conventional chemotherapeutic drugs will provide important information and improve our understanding of its potential in cancer therapy. 

## Figures and Tables

**Table 1:** IC_50_ values calculated on different cell lines after one cell-cycle treatment with *A. leucotrichus* extract.

Cell Line	IC_50_ (mg/mL)
HL60	0.354
TK6	0.223
DLD1	0.389
SHSY5Y	0.340

**Table 2:** Viability, RPD, apoptosis fold increase, and MNi fold increase on TK6 cells after 26 h treatments with *A. leucotrichus* at the indicated concentration compared to the untreated cultures. * *p* < 0.05 vs. untreated cultures.

	Viability %	RPD %	Apoptosis Fold Increase	MNi Fold Increase
Untreated cultures	100	100	1	1
*A. leucotrichus* 0.025 mg/mL	97.0 ± 1.7	87.5 ± 2.1	1.1 ± 0.1	1.1 ± 0.1
Perillaldehyde 0.025 mM	95.5 ± 1.0	83.2 ± 1.1 *	2.0 ± 0.1	0.9 ± 0.2

## Data Availability

The data presented in this study are available in article.

## References

[1] Greco G., Catanzaro E., Fimognari C. (2021). Natural Products as Inducers of Non-Canonical Cell Death: A Weapon against Cancer. Cancers.

[2] Turrini E., Catanzaro E., Muraro M.G., Governa V., Trella E., Mele V., Calcabrini C., Morroni F., Sita G., Hrelia P. (2018). *Hemidesmus Indicus* Induces Immunogenic Death in Human Colorectal Cancer Cells. Oncotarget.

[3] Turrini E., Calcabrini C., Tacchini M., Efferth T., Sacchetti G., Guerrini A., Paganetto G., Catanzaro E., Greco G., Fimognari C. (2018). In Vitro Study of the Cytotoxic, Cytostatic, and Antigenotoxic Profile of *Hemidesmus Indicus* (L.) R.Br. (Apocynaceae) Crude Drug Extract on T Lymphoblastic Cells. Toxins.

[4] Fimognari C., Lenzi M., Ferruzzi L., Turrini E., Scartezzini P., Poli F., Gotti R., Guerrini A., Carulli G., Ottaviano V. (2011). Mitochondrial Pathway Mediates the Antileukemic Effects of *Hemidesmus Indicus*, a Promising Botanical Drug. PLoS ONE.

[5] Lenzi M., Malaguti M., Cocchi V., Hrelia S., Hrelia P. (2017). *Castanea Sativa* Mill. Bark Extract Exhibits Chemopreventive Properties Triggering Extrinsic Apoptotic Pathway in Jurkat Cells. BMC Complement. Altern Med..

[6] Lenzi M., Cocchi V., Malaguti M., Barbalace M.C., Marchionni S., Hrelia S., Hrelia P. (2017). 6-(Methylsulfonyl) Hexyl Isothiocyanate as Potential Chemopreventive Agent: Molecular and Cellular Profile in Leukaemia Cell Lines. Oncotarget.

[7] Catanzaro E., Greco G., Potenza L., Calcabrini C., Fimognari C. (2018). Natural Products to Fight Cancer: A Focus on *Juglans Regia*. Toxins.

[8] Fimognari C., Berti F., Cantelli-Forti G., Hrelia P. (2005). Effect of Sulforaphane on Micronucleus Induction in Cultured Human Lymphocytes by Four Different Mutagens. Environ. Mol. Mutagen..

[9] Turrini E., Maffei F., Milelli A., Calcabrini C., Fimognari C. (2019). Overview of the Anticancer Profile of Avenanthramides from Oat. Int. J. Mol. Sci..

[10] Velasco-Negueruela A., Pérez-Alonso M.J., Pérez de Paz P.L., Palá-Paúl J., Sanz J. (2006). Analysis by Gas Chromatography–Mass Spectrometry of the Volatiles from the Fruits of *Ammodaucus Leucotrichus* Subsp. Leucotrichus and Subsp. Nanocarpus Grown in North Africa and the Canary Islands, Respectively. J. Chromatogr. A.

[11] Benhouhou S. (2005). *Ammodaucus Leucotrichus* Coss. &Dur. Guide to Medicinal Plants in North Africa.

[12] Sebaa A., Marouf A., Kambouche N., Derdour A. (2018). Phytochemical Composition, Antioxidant and Antimicrobial Activities of *Ammodaucus Leucotrichus* Fruit from Algerian Sahara. Orient. J. Chem..

[13] Merzouki A., Ed-derfoufi F., Molero Mesa J. (2000). Contribution to the Knowledge of Rifian Traditional Medicine. II: Folk Medicine in Ksar Lakbir District (NW Morocco). Fitoterapia.

[14] Hammiche V., Maiza K. (2006). Traditional Medicine in Central Sahara: Pharmacopoeia of Tassili N’ajjer. J. Ethnopharmacol..

[15] El-Mansouri L., Ennabili A., Bousta D. (2011). Socioeconomic Interest and Valorization of Medicinal Plants from the Rissani Oasis (SE of Morocco). Bol. Latinoamer Caribe Plantas Med. Arom.

[16] Jouad H., Haloui M., Rhiouani H., El Hilaly J., Eddouks M. (2001). Ethnobotanical Survey of Medicinal Plants Used for the Treatment of Diabetes, Cardiac and Renal Diseases in the North Centre Region of Morocco (Fez–Boulemane). J. Ethnopharmacol..

[17] Hmamouchi M., Heywood V.H., Skoula M. (1997). Plantes Alimentaires, Aromatiques, Condimentaires, Médicinales et Toxiques Au Maroc. Identification of Wild Food and Non-Food Plants of the Mediterranean Region.

[18] Nassif F., Tanji A. (2013). Gathered Food Plants in Morocco: The Long-Forgotten Species in Ethnobotanical Research. Life Sci..

[19] Beghalia M., Ghalem S., Allali H., Belouatek A., Marouf A. (2008). Inhibition of Calcium Oxalate Monohydrate Crystal Growth Using Algerian Medicinal Plants. J. Med. Plant Res..

[20] El-Ouady F., Eddouks M. (2020). Glucose Lowering Activity of Aqueous *Ammodaucus Leucotrichus* Extract in Diabetic Rats. Cardiovasc Hematol. Disord. Drug Targets.

[21] Bouknana S., Daoudi N.E., Bouhrim M., Ziyyat A., Legssyer A., Mekhfi H., Bnouham M. (2022). *Ammodaucus Leucotrichus* Coss. & Durieu: Antihyperglycemic Activity via the Inhibition of α-Amylase, α-Glucosidase, and Intestinal Glucose Absorption Activities and Its Chemical Composition. J. Pharm. Pharmacogn. Res..

[22] Gherraf N., Zellagui A., Kabouche A., Lahouel M., Salhi R., Rhouati S. (2017). Chemical Constituents and Antimicrobial Activity of Essential Oils of *Ammodaucus Leucotricus*. Arab. J. Chem..

[23] Louail Z., Kameli A., Benabdelkader T., Bouti K., Hamza K., Krimat S. (2016). Antimicrobial and Antioxidant Activity of Essential Oil of *Ammodaucus Leucotrichus* Coss. & Dur. Seeds. J. Mater. Environ. Sci..

[24] Selama G., Cadi H.E., Ramdan B., Majdoub Y.O.E., Dugo P., Mondello L., Cacciola F., Nhiri M. (2022). Determination of the Polyphenolic Content of *Ammodaucus Leucotrichus* Cosson and Durieu by Liquid Chromatography Coupled with Mass Spectrometry and Evaluation of the Antioxidant and Antiglycation Properties. J. Sep. Sci..

[25] Btissam R., Hafssa E.C., Mohamed N. (2022). Increased Non-Enzymatic Glycation Reported in *Apiaceae* Family Extracts. Pak. J. Bot..

[26] Dahmane D., Dob T., Krimat S., Nouasri A., Metidji H., Ksouri A. (2017). Chemical Composition, Antioxidant and Antibacterial Activities of the Essential Oils of Medicinal Plant *Ammodaucus Leucotrichus* from Algeria. J. Essent Oil Res..

[27] Khaldi A., Meddah B., Moussaoui A., Sonnet P. (2017). Anti-Mycotoxin Effect and Antifungal Properties of Essential Oil from *Ammodaucus Leucotrichus* Coss. & Dur. on *Aspergillus Flavus* and *Aspergillus Ochraceus*. J. Essent Oil-Bear Plants.

[28] Abu Zarga M.H., Al-Jaber H.I., Baba Amer Z.Y., Sakhrib L., Al-Qudah M.A., Al-humaidi J.Y.G., Abaza I.F., Afifi F.U. (2013). Chemical Composition, Antimicrobial and Antitumor Activities of Essential Oil of *Ammodaucus Leucotrichus* Growing in Algeria. J. Biol. Act Prod. Nat..

[29] Abdi Bellau M.L., Chiurato M.A., Maietti A., Fantin G., Tedeschi P., Marchetti N., Tacchini M., Sacchetti G., Guerrini A. (2022). Nutrients and Main Secondary Metabolites Characterizing Extracts and Essential Oil from Fruits of *Ammodaucus Leucotrichus* Coss. & Dur. (Western Sahara). Molecules.

[30] Wang C.Y., Wang S.Y., Chen C. (2008). Increasing Antioxidant Activity and Reducing Decay of Blueberries by Essential Oils. J. Agric. Food Chem..

[31] Tian J., Zeng X., Lü A., Zhu A., Peng X., Wang Y. (2015). Perillaldehyde, a Potential Preservative Agent in Foods: Assessment of Antifungal Activity against Microbial Spoilage of Cherry Tomatoes. Food Sci. Technol..

[32] Ito N., Nagai T., Oikawa T., Yamada H., Hanawa T. (2011). Antidepressant-like Effect of *l*-Perillaldehyde in Stress-Induced Depression-like Model Mice through Regulation of the Olfactory Nervous System. Evid. Based Complement. Alternat. Med..

[33] Catanzaro E., Turrini E., Kerre T., Sioen S., Baeyens A., Guerrini A., Bellau M.L.A., Sacchetti G., Paganetto G., Krysko D.V. (2022). Perillaldehyde Is a New Ferroptosis Inducer with a Relevant Clinical Potential for Acute Myeloid Leukemia Therapy. Biomed. Pharmacother..

[34] Lin Z., Huang S., LingHu X., Wang Y., Wang B., Zhong S., Xie S., Xu X., Yu A., Nagai A. (2022). Perillaldehyde Inhibits Bone Metastasis and Receptor Activator of Nuclear Factor-ΚB Ligand (RANKL) Signaling-Induced Osteoclastogenesis in Prostate Cancer Cell Lines. Bioengineered.

[35] Karlson J., Borg-Karlson A.-K., Unelius R., Shoshan M.C., Wilking N., Ringborg U., Linder S. (1996). Inhibition of Tumor Cell Growth by Monoterpenes in Vitro: Evidence of a Ras-Independent Mechanism of Action. Anticancer Drugs.

[36] Test No. 487: In Vitro Mammalian Cell Micronucleus Test. https://read.oecd-ilibrary.org/environment/test-no-487-in-vitro-mammalian-cell-micronucleus-test_9789264264861-en.

[37] Ahmed N., Williams J.F., Weidemann M.J. (1991). The Human Promyelocytic HL60 Cell Line: A Model of Myeloid Cell Differentiation Using Dimethylsulphoxide, Phorbol Ester and Butyrate. Biochem. Int..

[38] Kajstura M., Halicka H.D., Pryjma J., Darzynkiewicz Z. (2007). Discontinuous Fragmentation of Nuclear DNA during Apoptosis Revealed by Discrete “Sub-G1” Peaks on DNA Content Histograms. Cytometry.

[39] Honma M. (2020). An Assessment of Mutagenicity of Chemical Substances by (Quantitative) Structure–Activity Relationship. Genes Environ..

[40] Honma M., Kitazawa A., Kasamatsu T., Sugiyama K. (2020). Screening for Ames Mutagenicity of Food Flavor Chemicals by (Quantitative) Structure-Activity Relationship. Genes Environ..

[41] Witz G. (1989). Biological Interactions of α,β-Unsaturated Aldehydes. Free Radic. Biol. Med..

[42] Honma M., Yamada M., Yasui M., Horibata K., Sugiyama K., Masumura K. (2021). In Vivo and in Vitro Mutagenicity of Perillaldehyde and Cinnamaldehyde. Genes Environ..

[43] Elhajouji A., Van Hummelen P., Kirsch-Volders M. (1995). Indications for a Threshold of Chemically-Induced Aneuploidy in Vitro in Human Lymphocytes. Environ. Mol. Mutagen..

[44] Hernández L.G., van Benthem J., Johnson G.E. (2013). A Mode-of-Action Spproach for the Identification of Genotoxic Carcinogens. PLoS ONE.

[45] Enane F.O., Saunthararajah Y., Korc M. (2018). Differentiation Therapy and the Mechanisms That Terminate Cancer Cell Proliferation without Harming Normal Cells. Cell Death Dis..

[46] Kong G., Jeen Lee S., Jun Kim H., Surh Y.-J., Doo Kim N. (1999). Induction of Granulocytic Differentiation in Acute Promyelocytic Leukemia Cells (HL-60) by 2-(Allylthio) Pyrazine. Cancer Lett..

[47] Hou W., Wang Z.-Y., Lin J., Chen W.-M. (2020). Induction of Differentiation of the Acute Myeloid Leukemia Cell Line (HL-60) by a Securinine Dimer. Cell Death Discov..

[48] Martin S.J., Bradley J.G., Cotter T.G. (1990). HL-60 Cells Induced to Differentiate towards Neutrophils Subsequently Die via Apoptosis. Clin. Exp. Pharmacol. Immunol..

[49] Attaallah A., Lenzi M., Marchionni S., Bincoletto G., Cocchi V., Croco E., Hrelia P., Hrelia S., Sell C., Lorenzini A. (2020). A pro Longevity Role for Cellular Senescence. GeroScience.

[50] Lenzi M., Cocchi V., Hrelia P. (2018). Flow Cytometry vs Optical Microscopy in the Evaluation of the Genotoxic Potential of Xenobiotic Compounds. Cytom. Part B Clin. Cytom..

[51] Cocchi V., Hrelia P., Lenzi M. (2020). Antimutagenic and Chemopreventive Properties of 6-(Methylsulfinyl) Hexyl Isothiocyanate on TK6 Human Cells by Flow Cytometry. Front. Pharmacol..

[52] Lenzi M., Cocchi V., Cavazza L., Bilel S., Hrelia P., Marti M. (2020). Genotoxic Properties of Synthetic Cannabinoids on TK6 Human Cells by Flow Cytometry. Int. J. Mol. Sci..

[53] Cocchi V., Gasperini S., Hrelia P., Tirri M., Marti M., Lenzi M. (2020). Novel Psychoactive Phenethylamines: Impact on Genetic Material. Int. J. Mol. Sci..

[54] Lenzi M., Cocchi V., Gasperini S., Arfè R., Marti M., Hrelia P. (2021). Evaluation of Cytotoxic and Mutagenic Effects of the Synthetic Cathinones Mexedrone, α-PVP and α-PHP. Int. J. Mol. Sci..

[55] Ferruzzi L., Turrini E., Burattini S., Falcieri E., Poli F., Mandrone M., Sacchetti G., Tacchini M., Guerrini A., Gotti R. (2013). *Hemidesmus Indicus* Induces Apoptosis as Well as Differentiation in a Human Promyelocytic Leukemic Cell Line. J. Ethnopharmacol..

[56] Zhou J.Y., Norman A.W., Lübbert M., Collins E.D., Uskokovic M.R., Koeffler H.P. (1989). Novel Vitamin D Analogs That Modulate Leukemic Cell Growth and Differentiation with Little Effect on Either Intestinal Calcium Absorption or Bone Mobilization. Blood.

[57] Catino J.J., Miceli L.A. (1988). Microtiter Assay Useful for Screening of Cell-Differentiation Agents. J. Natl. Cancer Inst..

